# Association between sublingual microcirculation, tissue perfusion and organ failure in major trauma: A subgroup analysis of a prospective observational study

**DOI:** 10.1371/journal.pone.0213085

**Published:** 2019-03-05

**Authors:** Roberta Domizi, Elisa Damiani, Claudia Scorcella, Andrea Carsetti, Roberta Castagnani, Sara Vannicola, Sandra Bolognini, Vincenzo Gabbanelli, Simona Pantanetti, Abele Donati

**Affiliations:** Anaesthesia and Intensive Care, Department of Biomedical Sciences and Public Health, Università Politecnica delle Marche, Ancona, Italy; Duke University, UNITED STATES

## Abstract

**Introduction:**

Previous studies described impaired microvascular perfusion and tissue oxygenation as reliable predictors of Multiple Organ Failure in major trauma. However, this relationship has been incompletely investigated. The objective of this analysis is to further evaluate the association between organ dysfunction and microcirculation after trauma.

**Materials and methods:**

This is a retrospective subgroup analysis on 28 trauma patients enrolled for the Microcirculation DAIly MONitoring in critically ill patients study (NCT 02649088). Patients were divided in two groups according with their Sequential Organ Failure Assessment (SOFA) score at day 4. At admission and every 24 hours, the sublingual microcirculation was evaluated with Sidestream Darkfield Imaging (SDF) and peripheral tissue perfusion was assessed with Near Infrared Spectroscopy (NIRS) and Vascular Occlusion Test (VOT). Simultaneously, hemodynamic, clinical/laboratory parameters and main organ supports were collected.

**Results:**

Median SOFA score at Day 4 was 6.5. Accordingly, patients were divided in two groups: D4-SOFA ≤6.5 and D4-SOFA >6.5. The Length of Stay in Intensive Care was significantly higher in patients with D4-SOFA>6.5 compared to D4-SOFA≤6.5 (p = 0.013). Total Vessel Density of small vessels was significantly lower in patients with high D4-SOFA score at Day 1 (p = 0.002) and Day 2 (p = 0.006) after admission; the Perfused Vessel Density was lower in patients with high D4-SOFA score at Day 1 (p = 0.007) and Day 2 (p = 0.033). At Day 1, NIRS monitoring with VOT showed significantly faster tissue oxygen saturation downslope (p = 0.018) and slower upslope (p = 0.04) in patients with high D4-SOFA.

**Discussion:**

In our cohort of major traumas, sublingual microcirculation and peripheral microvascular reactivity were significantly more impaired early after trauma in those patients who developed more severe organ dysfunctions. Our data would support the hypothesis that restoration of macrocirculation can be dissociated from restoration of peripheral and tissue perfusion, and that microvascular alterations can be associated with organ failure.

## Introduction

In the last decades, advancements in prehospital and emergency hospital care led to a reduction in early mortality after multiple trauma. However, the occurrence of multi-organ failure (MOF) remains a major problem in severely injured patients and is responsible for delayed mortality, morbidity and prolonged stay in intensive care units [1,2].

Tissue damage and blood loss following trauma induce a systemic inflammatory response (SIRS) [3–5], with activation of complex haemostatic, inflammatory, endocrine and neurological processes that aggravate the initial damage caused (mainly) by hypoperfusion and reperfusion [6]. Cytokines and pro-inflammatory mediators are responsible for endothelial and glycocalyx dysfunction, increased capillary leakage, leukocyte activation and tissue oedema, which may lead to microcirculatory alterations similar to those observed during sepsis [2, 7–9]. An impairment in microvascular perfusion and tissue oxygenation has been described after traumatic haemorrhagic shock and was associated with higher risk of MOF and death [10–15]. Nonetheless, only a few studies investigated the pathophysiological and prognostic significance of microcirculatory dysfunction in patients with multiple trauma, both in the experimental and human settings [11–14].

Herein, we report a retrospective subgroup analysis of the single-centre prospective observational Microcirculation DAIly MONitoring in critically ill patients study (MicroDAIMON– NCT 02649088), in which we aimed to test the hypothesis that early alterations in microcirculation and tissue oxygenation are associated with the occurrence of organ failure in a heterogeneous cohort of adult patients admitted to the ICU for major trauma (major trauma defined as an Injury Severity Score being greater than 15) [16].

## Materials and methods

This is a retrospective subgroup analysis of the Microcirculation DAIly MONitoring in critically ill patients study (MicroDAIMON– NCT 02649088). The MicroDAIMON was a single-center prospective observational study where sublingual microcirculation and peripheral oxygenation measurements were performed in 97 adult critically ill patients (with respiratory, post-traumatic and general medical/surgical acute problems) on a daily basis from admission to ICU-discharge/death.

The study protocol was approved by the Local Ethics Committee (Comitato Etico Azienda Ospedaliero Universitaria Ospedali Riuniti Umberto I—G.M. Lancisi—G. Salesi of Ancona, currently named Comitato Etico Regione Marche—CERM; protocol number 212639, approval in date 14/02/2013) and it conformed to the principles of Helsinki declaration (last revision, Edinburgh 2000). A written informed consent was obtained, by signing the appropriate informed consent paperwork, from all the subjects or from their next of kin, in compliance with national applicable laws.

The aim of the study was to evaluate the association between microcirculatory alterations and outcome in a general ICU population.

Lack of written informed consent, age <18 years, pathophysiological conditions interfering with the acquisition of sublingual microcirculation videos (e.g. major maxillo-facial trauma) were exclusion criteria.

This retrospective analysis is focused on the subgroup of patients admitted in the ICU with a diagnosis of major trauma. The main objective of this analysis was to evaluate sublingual microcirculation and tissue perfusion after trauma and to assess if a correlation existed with the occurrence/persistence of organ failure, as identified by an elevated Sequential Organ Failure Assessment (SOFA) score. In a previous study by Tachon et al in patients with traumatic haemorrhagic shock, the SOFA score at day 4 after ICU admission was chosen to split the patients in high/low SOFA score groups, as ICU length of stay (LOS) in the two groups was significantly different [10]. Similarly, we divided the patients in two groups based on their SOFA score at day 4 (higher and lower or equal than the median value of SOFA score in the whole sample).

All patients were included in the study within the first 12 hours from ICU admission. At the moment of inclusion and every 24 hours thereafter, the sublingual microcirculation and peripheral tissue perfusion were evaluated. Simultaneously, arterial and central venous blood samples were withdrawn in order to assess blood gases, lactate levels and base excess. Baseline demographic data (age, sex, weight and height), hemodynamic parameters (Heart Rate, Mean Arterial Pressure, and Cardiac Output monitoring whenever available), clinical/laboratory data, and main organ supports were collected in digital excel spreadsheet.

Sublingual microcirculation was monitored with Sidestream DarkField (SDF) imaging technique (Microscan; Microvision Medical BV, Amsterdam, The Netherland).

Extensive details on the SDF imaging technique have been described in previous papers, however the below gives a brief summary of the technique. Microscan is equipped with a ring of green light-emitting diodes (LEDs) located at the end of a probe. The LEDs light of Microscan is characterized by a specific wavelength of 530 nm that is absorbed by the haemoglobin contained in red blood cells (RBC) so that RBC are visualized as flowing granules that indirectly highlight just those vessels that are perfused, hiding vessels that are not perfused [17,18]. We tried to obtain the highest quality of picture by avoiding pressure and movement artefacts, improving focus and illumination and cleaning the sublingual mucosa from saliva and blood. Videos from at least 5 different sites of sublingual microcirculation were recorded for each session, and the best 3 of them were analysed offline using a dedicated software package (Automated Vascular Analysis Software; Microvision Medical BV).

Microcirculatory parameters were calculated offline with the Automated Vascular Analysis software (AVA v3, Microvision Medical, Amsterdam, NL), according to the 2007 round table conference and “the microcirculation image quality score”: Massey et al [17,19].

The analysis of microvascular flow focused on vessels with a diameter of 0–20 μm (small-size vessels). Medium-size vessels (diameter 20–50 μm), that consist in precapillary arterioles and postcapillary venules, were used as quality index and as tool to identify pressure artefacts and mechanical occlusion to flow (small vessels with disturbed microcirculatory flow along with flow disruptions in medium vessels) [17–19].

Total vessel density (TVD) and perfused vessel density (PVD), De Backer score, the proportion of perfused vessels (PPV) and the microvascular flow index (MFI), were calculated for small-size vessels (diameter ≤ 20 μm) in all the videos analysed.

Peripheral skeletal muscle tissue oxygenation was evaluated using Near-InfraRed Spectroscopy (InSpectra Model 650; Hutchinson Technology Inc., Hutchinson, MN, USA) with a 15-mm probe on the thenar eminence. A vascular occlusion test (VOT) was performed in order to assess the variations in tissue oxygen saturation (StO2) during a transient ischemic challenge.

A sphygmomanometer cuff was placed around the forearm and kept inflated to 50 mm Hg above the systolic arterial pressure until the StO2 reached 40%. The StO2 downslope (%/minute) was calculated from the regression line of the StO2 decay during vascular occlusion and provides an index of O2 extraction and consumption rate. The StO2 upslope (%/minute) was calculated from the regression line of the reperfusion phase and reflects microvascular reactivity, capillary recruitment and post-ischemic vasodilatation. The area under the curve (AUC) of StO2 represents the hyperaemic response [20]. (S1 Fig).

The Kolmogorov-Smirnov test was check for normality of distribution of continuous variables. Since most of the parameters showed a non-normal distribution, data are presented as median (and InterQuartile range, IQR) or as N° (and %) for nominal variables. Non-parametric tests (Mann–Whitney and χ2 with Fisher’s exact tests) were used as appropriate for comparison between independent samples. Differences were considered significant at P values of less than 0.05 (two-sided).

The area under the Receiver Operating Characteristic (ROC) curve was calculated to evaluate the discriminative ability of microvascular parameters and NIRS variables towards SOFA score at day 4 (D4-SOFA).

Statistical analysis was performed using the Statistical Package for Social Science software, version 17.0 (SPSS Inc).

The study protocol was approved by the Local Ethics Committee and it conformed to the principles of Helsinki declaration (last revision, Edinburgh 2000). A written informed consent was obtained, by signing the appropriate informed consent paperwork, from all the subjects or from their next of kin, in compliance with national applicable laws.

## Results

Of the cohort of 97 patients enrolled in the MicroDAIMON study, 39 were admitted with a diagnosis of multiple trauma.

Median age was 55 (35–74) years, 30 patients (77% of total) were male, most of them were previously healthy (71% with less than two comorbidities at admission in ICU) and the leading causes of trauma were road collisions. Head-and-neck and chest were injured in most of the patients. 18 patients (46%) were transfused in the Emergency Room and 18 (46%) received surgery before admission in ICU (S1 Table). Median values for Apache score at admission in ICU was 14 (7–18) and for SOFA score was 7 (4–9); the 87% of the patients were haemodynamically stable at admission in ICU.

Median ICU length of stay (ICU-LOS) was 7 days (4–15), with a hospital mortality of 20.5% (8 patients).

Three of the 39 patients deceased in ICU (7.7%) within 72 hours from admission, 5 patients were discharged from ICU before Day 4, three further patients missed data for SOFA score calculation at day 4 and they were excluded a priori. Therefore, this analysis includes 28 patients in total.

Median SOFA score at Day 4 was 6.5 (4–9). Accordingly, patients were divided in two groups: D4-SOFA ≤6.5 and D4-SOFA >6.5.

ICU-LOS was significantly higher in patients with D4-SOFA>6.5 with a median of 15 (9–25) versus 7 (4–13) days for patients with D4-SOFA≤6.5 (p = 0.013). Patients with high SOFA score at Day 4 were younger than patients with lower SOFA score: 44 (29–76) versus 69 years (41–74) years, but the difference wasn’t statistically significant (p = 0.458). The admission SOFA score was higher in those patients who had D4-SOFA score> 6.5 (8 [7–9.5] versus 5 [3–9]; p = 0.037).

In the first 4 days, the two groups did not differ for Mean Arterial Pressure (MAP), Heart Rate (HR), Lactate, Central Venous Saturation (ScVO2), haemoglobin and RBC transfusion requirements (Table 1).

**Table 1:** Hemodynamic variables in the first four days of ICU admission for the two groups of patients (SOFA^a^ score at D4≤6.5 and SOFA score at D4>6.5).

		*SOFA≤6*.*5 at D4*	*SOFA>6*.*5 at D4*	*p value*
		15 (53.6%)	13 (46.4%)	
***MAP***^b^**, *mmHg***				
	D1	86 [75–99]	85 [77–95]	0.964
	D2	82 [71–99]	89 [71–95]	0.648
	D3	89 [84–102]	88 [79–97]	0.413
	D4	91 [74–106]	88 [84–89]	0.932
***HR***^c^**, *bpm***				
	D1	63 [57–83]	83 [74–102]	0.142
	D2	77 [64–100]	84 [60–101]	0.717
	D3	71 [62–88]	78 [63–92]	0,964
	D4	75 [64–87]	74 [70–85]	0.843
***Norepinephrine*, *mcg/kg/min***				
	D1	0.00 [0.00–0.22]	0.23 [0.026–0.48]	***0*.*033***
	D2	0.00 [0.00–0.182]	0.22 [0.02–0.28]	0.085
	D3	0.00 [0.00–0.00]	0.09 [0.04–0.33]	***0*.*001***
	D4	0.00 [0.00–0.00]	0.1 [0.04–0.17]	***0*.*001***
***Arterial pH*, *AU***				
	D1	7.43 [7.4–7.58]	7.45 [7.38–7.49]	0.329
	D2	7.48 [7.44–7.49]	7.45 [7.42–7.49]	0.892
	D3	7.47 [7.43–7.52]	7.48 [7.43–7.53]	0.440
	D4	7.48 [7.44–7.49]	7.47 [7.45–7.51]	0.936
***BE***^d^**, *mmol/L***				
	D1	0.6 [(-)0.9,-2.3]	0.6 [(-)2.7–3.8]	0.880
	D2	4.5 [2.2–8.5]	4.4 [1.7–6.2]	0.751
	D3	5.5 [3.7–9.4]	6.4 [2.1–7.6]	0.892
	D4	5.2 [3.7–9.7]	7.7 [4.2–11.8]	0.538
***Lactate*, *mmol/L***				
	D1	1.3 [0.9–2.0]	1.7 [1.1–2.2]	0.235
	D2	1.0 [0.7–2.0]	1.1 [0.7–2.0]	0.217
	D3	0.9 [0.8–1.1]	0.9 [0.7–1.2]	0.413
	D4	0.7 [0.7–1.1]	0.9 [0.6–1.4]	0.590
***ScvO2***^e^**, *%***				
	D1	79.4 [75.6–81.7]	74.3 [59.2–84.4]	0.161
	D2	74.8 [67.1–80.8]	79.3 [70.7–85.2]	0.347
	D3	75 [67.5–78.6]	72 [71.6–90.6]	0.219
	D4	72.1 [68.6–74.4]	78.6 [73.3–81.0]	0.148
***Hb***^f^**, *g/dl***				
	D1	10.8 [9.0–12.4]	10.4 [10.2–11.2]	0.751
	D2	10.3 [9.2–11.5]	9.3 [8.4–9.9]	0.170
	D3	9.3 [8.9–11.4]	9.8 [9.5–10.9]	0.316
	D4	9.4 [8.2–11.3]	9.8 [9.3–10.8]	0.630
***Hct***^g^**, *%***				
	D1	30.2 [27.1–3.8]	30.7 [29.2–32.]	0.856
	D2	30.9 [26.0–34.3]	27.4 [24.4–29.7]	0.170
	D3	27.7 [27.0–34.8]	29.5 [28.6–31.4]	0.440
	D4	29.0 [25.8–33.9]	29.6 [27.8–31.9]	0.79
***Patients transfused*, *n° (%)***				
	D1	7 (47)	3 (23)	0.254
	D2	0 (0)	5 (39)	***0*.*013***
	D3	2 (13)	3 (23)	0.639
	D4	3 (25)	2 (17)	0.99

Hemodynamic data were collected daily, at the same time of microcirculatory assessment. Data presented as median [IQR] or number [%].

^a^SOFA = Sequential Organ Failure Assessment;

^b^MAP = Mean Arterial Pressure;

^c^HR = Heart Rate;

^d^BE = Base Excess;

^e^ScVO2 = Central venous Saturation

^f^Hb = Haemoglobin;

^g^Hct = Haematocrit,

**Fig 1:**
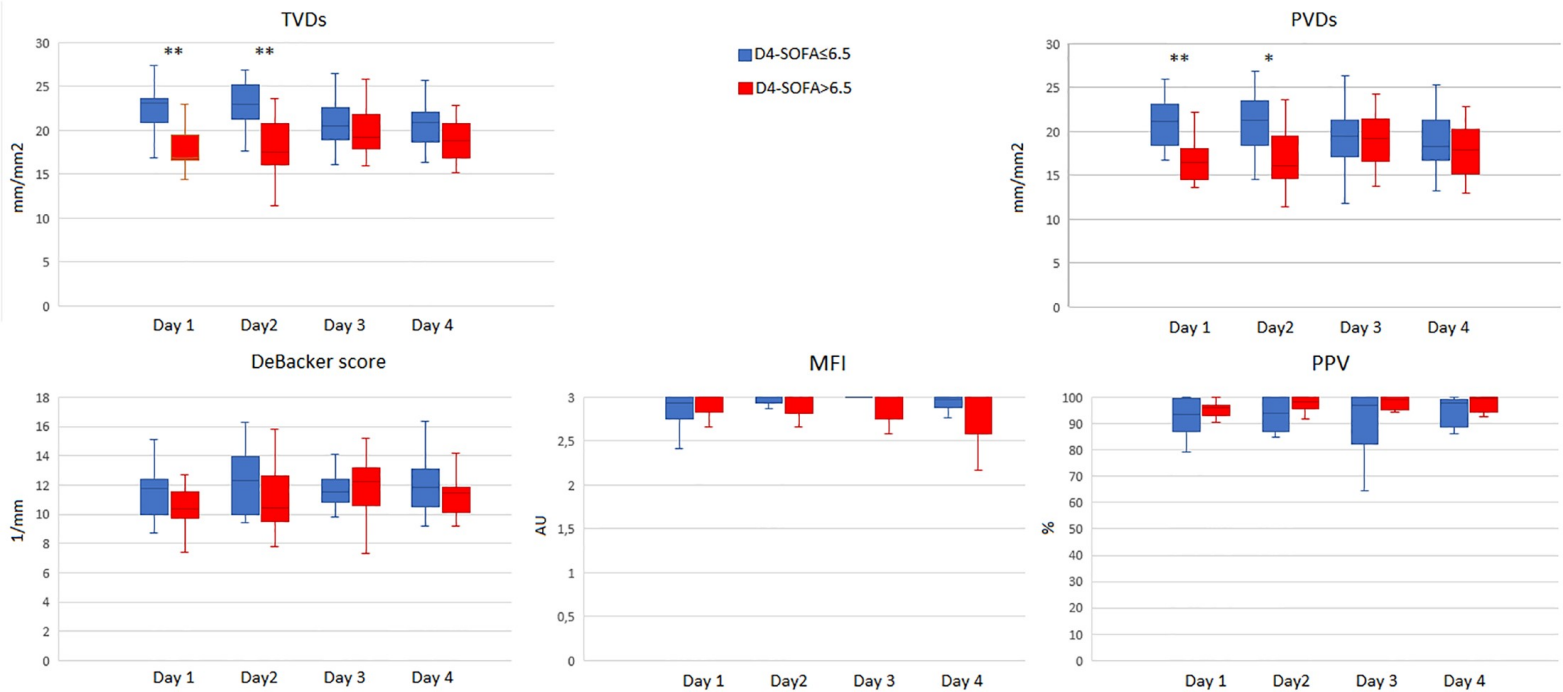
Changes in the sublingual microvascular parameters in the first 4 days of admission in ICU for the two groups of patients (SOFA score at D4≤6.5 and SOFA score at D4>6.5). * p<0.05; ** p<0.01. TVDs: total small vessel density; PVDs: perfused small vessel density; MFI: microvascular flow index; PPV: percentage of perfused vessels.

Norepinephrine infusion was significantly higher in high SOFA group at Day 1, Day 3 and Day 4. Total Vessel Density of small vessels (TVDs) was significantly lower in patients with D4-SOFA >6.5 both at Day 1 (17.33 [16.58–21.63] versus 23.24 [18.51–23.72] mm/mm2; p = 0.002) and at Day 2 (17.45 [15.82–22.51] versus 22.92 [20.64–25.58] mm/mm2, p = 0.006); Perfused Vessel Density of small Vessels (PVDs) showed similar results at Day 1 and Day 2 with lower PVDs in patients with D4-SOFA score>6.5 (16.55 [17.94–23.93] versus 20.91 [17.57–23.45] mm/mm2 at Day 1, p = 0.007; 16.52 [14.24–19.92] versus 21.32 [17.94–23.93] mm/mm2 at Day 2, p = 0.033 (Fig 1).

MFI and PPV did not show any difference between the groups, De Backer score tended to be lower in the high SOFA score group at Day 1 and Day 2 although the difference was not significant (Fig 1).

**Fig 2:**
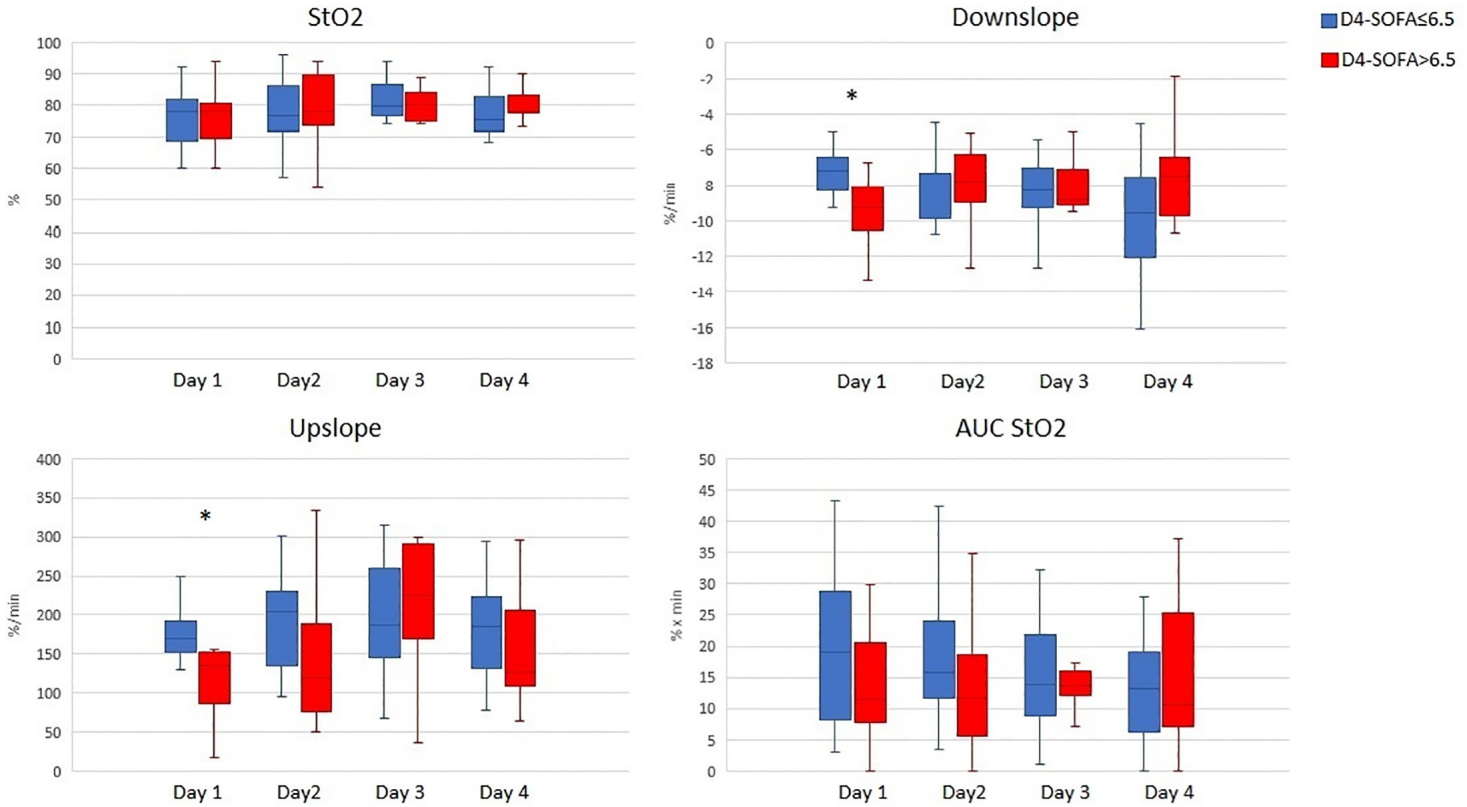
Changes in NIRS-derived parameters in the first 4 days of admission in ICU for the two groups of patients (SOFA score at D4≤6.5 and SOFA score at D4>6.5). * p<0.05. StO2: tissue oxygen saturation; AUC StO2: area under the curve of tissue oxygen saturation.

Changes in NIRS-derived parameters in the two groups are shown in Fig 2.

StO2 was similar in the first 4 days between patients with lower and higher D4-SOFA. At Day 1 desaturation was faster for patients with D4-SOFA> 6.5 with a median StO2 downslope of -9.3 (-11.6, —7.5) versus -7.12 [IQR (-8.5,-6.2] %/minute for patients with D4-SOFA≤6.5 (p = 0.018). The StO2 upslope was lower in high-SOFA patients at D1 (141 [75–190] versus 170 [150–193] %/min in low-SOFA patients (p = 0.04). Similar results were evident at D2, however the difference was not statistically significant (Fig 2).

**Fig 3:**
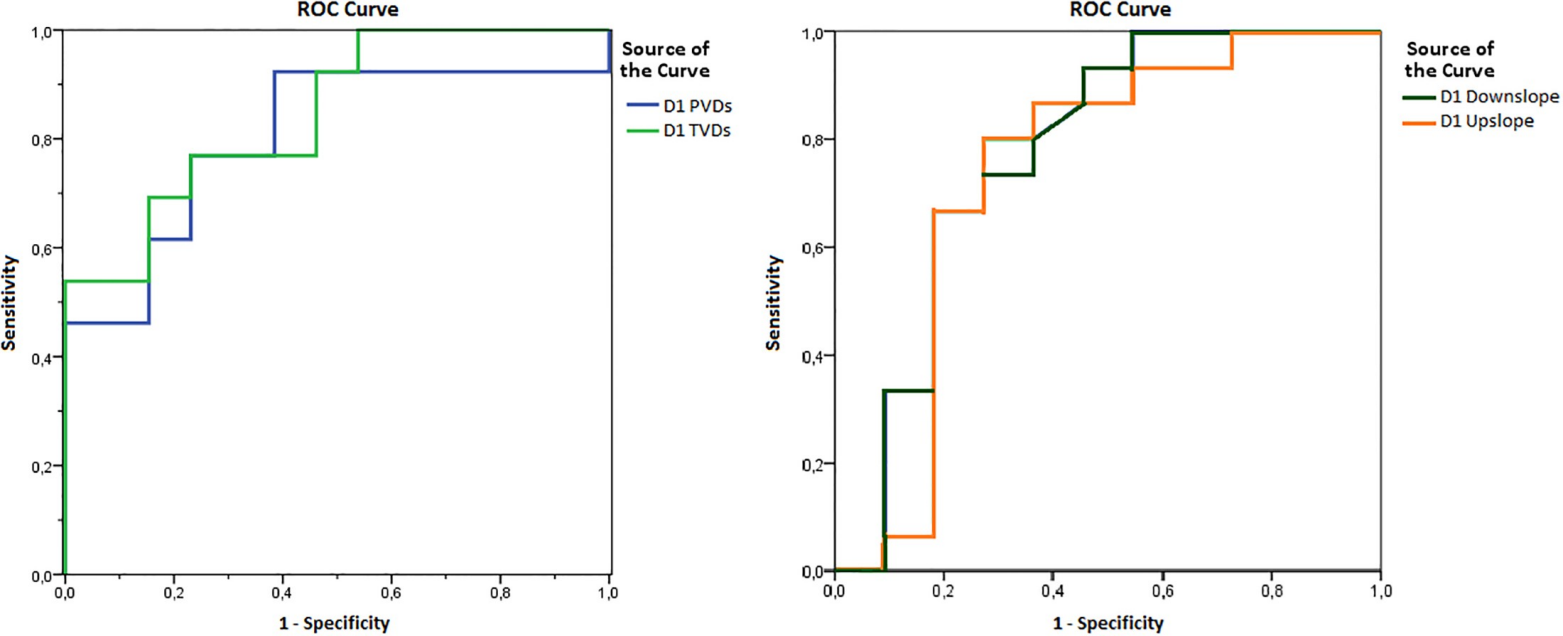
Receiver Operating Characteristic (ROC) curves. Discriminative ability of PVDs and TVDs (on the left) and StO2 downslope and upslope (on the right) at admission in ICU towards D4 SOFA score. TVDs: total small vessel density; PVDs: perfused small vessel density. StO2: tissue oxygen saturation.

ROC curve analysis showed that low PVDs and TVDs at admission in ICU were significant predictors of D4-SOFA score >6.5 with an Area Under the curve of 0.805 for PVDs (95% confidence interval [CI] 0.627–0.983, p = 0.08) and AUC of 0.85 for TVDs (95% CI = 0.699–0.993; p = 0.03) (Fig 3).

The StO2 Downslope and StO2 Upslope at D1 were also able to predict a higher D4-SOFA (AUC 0.773 [95%CI 0.57–0.97] p = 0.02 and 0.74 [95%CI 0.52–0.96] p = 0.04, respectively).

## Discussion

In this retrospective analysis of prospectively collected data on 28 patients with major trauma, patients with higher SOFA score at day 4 (D4-SOFA>6.5) after admission in ICU showed significantly lower sublingual microvascular density in the first two days in the ICU as compared to those with a SOFA score ≤6.5 at day 4, while no differences were observed in parameters of microcirculatory blood flow quality. In addition, a higher peripheral tissue oxygen extraction rate and impaired microvascular reactivity at admission were associated with a SOFA score >6.5 at day 4. These microcirculatory disturbances occurred despite early haemodynamic stabilization, as suggested by similar values of MAP, HR, lactate and ScvO2 in the two groups, although patients with a D4-SOFA>6.5 required higher doses of norepinephrine in the first 4 days.

Our results are consistent with data from previous literature. The microvascular response to major trauma was studied from Tachon et al that demonstrated that disturbances in the microcirculation in the first 72 hours after ICU admission were associated with organ failure and with longer ICU stay in their cohort of traumatic haemorrhagic shock. However, differently from our results, MFI and PPV were significantly different between the groups and Functional Capillary Density was assessed instead of PVD and TVD [10]. The two studies can be easily compared as Perfused Vessel Density (PVD) is an estimate of Functional Capillary Density (FCD), calculated by multiplying vessel density by the proportion of perfused vessels.

Gòmez et al performed NIRS measurements and evaluated the response to a VOT in ICU trauma patients and healthy controls. They showed slower StO2 recovery upslopes in trauma patients than in controls, with similar downslope curves, suggesting a local reduction in microcirculatory reserve, however microcirculatory flow distribution was not assessed in this study [21].

Duret et al showed significantly altered StO2 and desaturation slopes at admission were associated with no improvement or worsening organ failure in the first 72 hours in patients with traumatic haemorrhagic shock [22]. In the present study, StO2 was similar at all timepoints between the two groups, suggesting that StO2 alone may not be an adequate predictor of tissue hypoperfusion at least in some patient categories, and that the ischaemic challenge derived from VOT may be more sensitive to highlight signs of altered tissue perfusion and oxygenation [23].

Although the retrospective design of our analysis does not allow to speculate on potential confounding factors, our data suggest that a reduction in microvascular perfusion and reactivity in the first hours after a multiple trauma may predispose patients to the development of tissue oxygen deficit and organ failure.

Impaired tissue perfusion and reduced microvascular density may be considered markers of insufficient fluid resuscitation and persistent low-grade hypovolaemia: microvascular disturbances have been previously showed in hypovolaemic shock and are characterized by deterioration of Functional Capillary Density and reduction of TVD [24–25]. Fluid resuscitation can cause an apparent improvement in systemic macrocirculation, while leaving inadequate regional oxygenation and microvascular perfusion. This pattern was demonstrated both in hypovolaemic and haemorrhagic shock that are the main features of early traumatic shock [24–28]

Afterwards, trauma may induce further mechanisms of complex microvascular impairment: the extensive tissue damage and tissue hypoperfusion may induce release of inflammatory mediators and reactive oxygen species and it can produce Systemic Inflammatory Response Syndrome with deterioration of the endothelial function and of glycocalyx, alterations in red blood cell deformability and increase in leukocyte adhesion. Any one of these alterations either alone or acting together can lead to a more distributive shock characterized by a loss of microvascular integrity, reduction of capillary density, increase in vascular permeability and interstitial oedema. The enhanced oxygen diffusion distance can result in tissue hypoxemia and organ dysfunction.

We can suggest that in severe trauma patients, as in sepsis and other patterns of shock a loss of coherence between macro and microhemodynamic exists, and medical interventions aimed at the correction of systemic hemodynamic variables may fail to be effective in correcting regional and microcirculatory perfusion and oxygen delivery to the parenchymal cell. [27].

Finally, the patients with D4-SOFA>6.5 received higher norepinephrine doses than the group with a SOFA score ≤6.5 at day 4. We cannot exclude a direct relationship between vasopressor use and the observed microcirculatory alterations, because high dose norepinephrine, together with inadequate fluid resuscitation, can induce excessive vasoconstriction and capillary de-recruitment and increasing MAP with norepinephrine showed no impact on improvement of microcirculatory perfusion in different patterns of shock. [28–32] However, while norepinephrine dose was consistently different in the two groups even at Day 3 and Day 4 post-admission in ICU, microcirculatory alterations were not evident after Day 2, supporting the hypothesis that vasopressor use alone cannot explain the microvascular pattern demonstrated early after ICU admission. Unfortunately, our study is not powered to answer these questions.

Nevertheless, as in our patients a persistent microcirculatory under-resuscitation in the presence of normalized systemic hemodynamic was associated with adverse clinical outcome, this study suggests that early identification of microvascular and tissue hypoperfusion in trauma patients may be relevant in the clinical practice to detect patients at higher risk to develop multiple organ failure and to guide the clinician to a tailored intensive care treatment in term of fluids and/or vasoactive drugs. Moreover, further studies on this specific field will help us to understand if a more accurate resuscitation, titrated on bedside assessment of microcirculation instead of hemodynamic alone would be important in the intensive care treatment of major trauma.

Our study has several limitations. First, the retrospective design did not allow to control for potential confounders. Second, this was a subgroup analysis of a prospective observational study with a different primary goal: some of the analyses may thus be underpowered to detect statistically significant differences. In addition, the small sample size prevented evaluate of the relationship between microvascular alterations and other outcomes, such as mortality. Third, the observational design of our study does not allow us to clarify whether the implementation of microcirculation-targeted therapies may be able to modify the outcome and prevent the development of organ dysfunctions. Fourth, the lack of cardiac output monitoring impeded a more comprehensive overview of the haemodynamic state and of its relationship with microvascular perfusion.

## Conclusion

In our cohort of patients with major trauma, sublingual microcirculation and peripheral microvascular reactivity were significantly impaired among those patients who then showed more severe organ dysfunction. Our data suggest that early impairment in microvascular perfusion after severe trauma may be associated with development of organ failure. Our study would support the hypothesis that restoration of macrocirculation can be dissociated from restoration of peripheral and tissue perfusion.

Therefore, evaluating the microcirculation together with the macrohemodynamic variables in this patient category may represent a tool to identify those patients with higher risk of MOF, who could benefit from closer monitoring and additional therapeutic efforts. Further studies are needed to clarify the role of microvascular dysfunction in the pathophysiology of MOF after multiple trauma.

## Supporting information

S1 Fig Near-InfraRed Spectroscopy (NIRS), StO2 response to vascular occlusion test (VOT). (TIF)

S1 Table Baseline characteristics of the 39 trauma patients included in MicroDAIMON study; n (%). ^a^ER = Emergency Room.
(PDF)

S2 Table Supporting information; anonymized datasheet with main data collected. SOFA = Sequential Organ failure Score; PVDs = perfused small vessel density; TVDs = total small vessel density; PPV = percentage of perfused vessels; MFI = microvascular flow index; StO2 = tissue oxygen saturation; AUC StO2 = area under the curve of tissue oxygen saturation; LOS = Length of Stay; ICU = Intensive Care Unit; APACHE = Acute Physiology, Age, Chronic Health Evaluation; NE = Norepinephrine; HR = Heart Rate; MAP = Mean Arterial Pressure; Hb = Haemoglobin.
(PDF)
