# Supplementary figures and images for: Association between sublingual microcirculation, tissue perfusion and organ failure in major trauma: A subgroup analysis of a prospective observational study
Source: PLoS One. 2019 Mar 5;14(3):e0213085. doi: 10.1371/journal.pone.0213085 (PMC6400441; doi:10.1371/journal.pone.0213085)

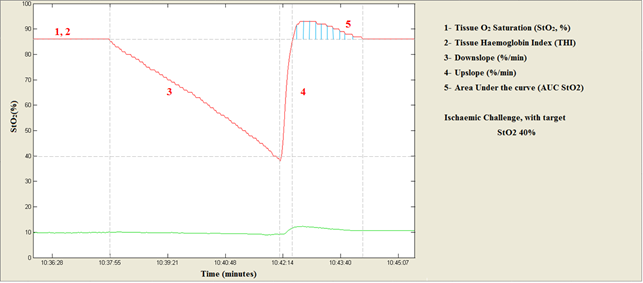

Supplement: S1 Fig — (TIF) [file pone.0213085.s001.tif]
