# Supplementary material for: Association between sublingual microcirculation, tissue perfusion and organ failure in major trauma: A subgroup analysis of a prospective observational study
Source: PLoS One. 2019 Mar 5;14(3):e0213085. doi: 10.1371/journal.pone.0213085 (PMC6400441; doi:10.1371/journal.pone.0213085)
Supplement: S1 Table — aER = Emergency Room. (PDF) [file pone.0213085.s002.pdf]

S1 Table: Baseline characteristics of the 39 trauma patients included in MicroDAIMON study; n (%).

**Mechanism of trauma, n(%)**		**n (%)**
	Traffic	20 (51.3%)
	Fall	9 (23.1%)
	Knife wound	3 (7.7%)
	Other	7 (17.9%)
**Injured area, n(%)**		
	Head and neck	19 (48,7%)
	Face	8 (20,1%)
	Chest	19 (48,7%)
	Abdomen	15 (38,5%)
	Extremity	12 (30,1%)
	Other	7 (17,9%)
**Crush Syndrome, n(%)**		
		5 (12.8%)
**Patients transfused in the ER[a]**		
		18 (46%)
**Surgery after ER**		
		18 (46%)

[a]ER=Emergency Room
