# Supplementary material for: Association between sublingual microcirculation, tissue perfusion and organ failure in major trauma: A subgroup analysis of a prospective observational study
Source: PLoS One. 2019 Mar 5;14(3):e0213085. doi: 10.1371/journal.pone.0213085 (PMC6400441; doi:10.1371/journal.pone.0213085)
Supplement: S2 Table — SOFA = Sequential Organ failure Score; PVDs = perfused small vessel density; TVDs = total small vessel density; PPV = percentage of perfused vessels; MFI = microvascular flow index; StO2 = tissue oxygen saturation; AUC StO2 = area under the curve of tissue oxygen saturation; LOS = Length of Stay; ICU = Intensive Care Unit; APACHE = Acute Physiology, Age, Chronic Health Evaluation; NE = Norepinephrine; HR = Heart Rate; MAP = Mean Arterial Pressure; Hb = Haemoglobin. (PDF) [file pone.0213085.s003.pdf]

S2 Table: Supporting information; anonymized datasheet with main data collected.

SOFA_admission	SOFA_D2	SOFA_D3	SOFA_D4	PVD_D1	TVD_D1	PPV_D1	DeBacker_D1	MFI_D1	StO2_D1	Downslope_D1	Upslope_D1	AUC_D1	PVD_D2	TVD_D2	PPV_D2	DeBacker_	MFI_D2
13	13	11	12	18,86	19,44	96,79	12,27	2,92	75	-9,25	121,5	11,5	14,01	14,01	100	9,18	3
10	12	10	11	16,03	16,58	96,78	8,89	2,83	64	-3,72	17,35	0	19,32	19,91	96,84	12,47	2,75
8	9	8	10	22,16	22,96	96,44	12,68	2,53	80	-8,34	67,28	7,1	23,46	23,46	100	13,31	3
7	10	7	10	16,52	16,63	99,38	9,92	3	60	-9,83	134,57	10,8	16,31	16,88	96,83	10,83	3
11	8	8	9	13,58	14,44	94,62	9,71	3					16	16,46	97,08	9,66	2,83
9	9	10	9	14,21	16,58	86,18	7,41	3					13,89	18,58	75,93	10,03	3
8	8	8	9	18,02	18,02	100	10,78	3	78	-7,76	155,51	14,6	18,04	18,04	100	11,95	3
7		9	9	14,55	15,01	95,93	10,12	3	81	-11,19	540	28,9					
5	10	9	9	16,07	16,84	94,89	11,29	2,42	94	-9,68	294,64	8,6	14,94	16,23	91,73	10,05	2,42
7	8	8	7	22,12	22,36	98,97	11,54	2,92	78	-6,71	146,55	26,3	23,65	23,65	100	13,02	3
7	7	7	7	16,59	17,84	92,98	10,32	3	78	-13,37	94,2	12,6	11,4	11,4	100	7,81	3
9	9	7	7	13,77	15,05	90,4	9,52	2,67	81	-13,04	77,32	6,9	15,58	15,69	99,1	8,85	2,67
5	9	9	9	17,62	22,66	77,39	16,01	3	64	-8,88	149,64	29,9	20,13	23,38	86,4	15,8	3
10	8	7	6	25,9	28,32	91,38	16,68	2,58	71	-6,62	149,41	7,3	24,14	25,02	96,32	16	2,92
13	12	14	6	18,47	23,37	79,15	11,83	2,33	67	-7,61	192,86	23,7	14,49	21,58	66,23	11,41	3
6	5	6	6	17,28	17,63	97,82	9,05	2,75	60	-9,07	175	43,2	23,31	23,31	100	13,14	3
4	7	8	5	23,12	23,12	100	13,87	3	61	-7,17	84	15,9	25,77	25,77	100	14,68	3
5	5	3	5	11,21	17,72	63,35	11,72	2,92	92	-5,84	191,79	21,9	17,71	20,33	87,16	13,44	3
9	10	6	4	20,64	20,88	98,25	10,48	2,93	77,9	-8,52	170	9	16,92	17,68	95,78	10,88	2,87
3	7	4	4	16,72	16,83	99,35	9,42	3	70	-8,88	129,64	6,8	19,27	19,27	100	9,84	2,93
4	8	8	4	22,27	22,37	99,54	12,17	2,93	81	-6,35	174,64	3,2					
3	3	3	4	23,8	27,45	86,8	8,71	3	82	-7,19	150,27	21,6	23,19	26,38	87,85	9,73	3
8	8	3	3	21,18	23,64	88,39	15,12	2,42	73,3	-5,42	165,71	33,7	20,95	22,54	92,27	13,7	2,5
9	8	5	3	23,56	23,56	100	12,35	3	88	-4,94	249	3	26,92	26,92	100	16,29	3
1	3	2	2						81,2	-9,28	152,45	11,2					
0	0	1	1						89,2	-8,14	306,86	19					
2	2	2	2	18,71	22,37	83,83	9,97	3	68	-6,92	228	495	21,7	24,98	86,94	10,05	3
5	4	4	4	22,24	23,75	93,68	10,38	3	81	-7,3	168,73	37,3	18,64	21,99	84,78	9,41	3

StO2_D2	Downslop	Upslope_	AUC_D2	PVD_D3	TVD_D3	PPV_D3	DeBacker	_MFI_D3	StO2_D3	Downslop	Upslope_	AUC_D3	PVD_D4	TVD_D4	PPV_D4	DeBacker	_MFI_D4
58	-9,11	50,36	6,5	21,86	21,86	100	13,61	2,75					22,87	22,87	100	13,39	2,92
74	-5,01	92,73	10,7	15,08	15,97	94,27	9,54	3	60	-4,94	36,47	15,4	13,02	16,58	77,56	11,06	1,5
94	-10	333,43	5,7	24,26	24,46	99,23	14,74	3	80	-8,83	164	16,5	22,07	22,07	100	11,79	3
78	-7,74	137,14	4,8	18,61	19,24	96,75	12,45	2,58	82	-7,11	176,43	13,7	18,01	18,96	95,02	11,58	2,17
				16,06	16,24	98,79	9,27	2,58					15,21	15,21	100	9,32	3
54	-5,97	102	16,6	13,75	18,54	73,54	7,32	3	74	-5,04	58,22	15,1	14,13	18,81	74,73	9,16	3
78	-6,37	77,25	12,6	19,93	20,03	99,49	12,19	3	83	-9,14	266,57	7,2	20,04	20,43	98,63	11,79	3
81	-8,53	399	34,9	19,2	19,2	100	12,11	3	75	-8,04	294	13,5	17,71	17,71	100	11,94	3
94	-7,82	182,41	0	16,62	17,32	95,15	11,76	2,67	80	-9,48	286,29	17,4	20,98	21,58	96,77	14,19	2,58
73	-6,76	207	24,4	23,73	23,73	100	13,12	3	87	-9,02	296,57	10,2	19,58	19,58	100	11,38	3
89,9	-6	147,86	13,9	19,48	19,48	100	13,02	3	88,7	-8,13	300	13,6	17,02	17,02	100	10,18	3
89,5	-12,66	72,33	4,9	17,91	17,91	100	10,56	3	85	-9,09	225	10,7	15,15	16,3	92,57	10,01	2,58
77,8	-8,94	64,39	31	21,39	25,84	82,55	15,19	3	75	-9,41	211,07	24,8	21,39	25,84	82,55	15,19	3
76,8	-10,76	134	11,7	11,79	18,07	64,49	11,49	1,33	74,1	-10,17	187,5	19,6	25,37	25,65	98,8	16,36	2,92
71,9	-16,48	136,07	12,5	21,52	26,54	81,26	12,43	3	76	-8,22	114,23	10,6	13,21	19,42	68,17	9,22	2
73	-9,61	204,64	29,3	7,94	7,94	100	6,47	3	92	-9,11	67,9	6,5	16,18	16,49	98,48	10,61	2,92
73	-8,01	102,55	15,7	21,71	21,71	100	12,38	3	77	-6,59	174,64	20,5	18,74	19,13	97,98	11,5	3
85	-7,25	222,86	34,3	16,16	19,29	84,21	13,72	3	83	-8,42	255,43	29,5	18,38	21,43	86,3	12,85	3
79,8	-8,56	234	4	18,04	18,65	96,11	9,82	3	79,4	-5,52	213,21	23,1	16,92	17,31	97,62	10,18	2,77
57,3	-4,44	97,14	19	20,55	20,55	100	10,78	3	78,5	-7,64	264	6,5	21,33	21,33	100	11,89	2,93
88,6	-10,06	270	11,3	21,56	21,66	99,58	13,38	3	83	-9,47	96,73	13,9	23,95	23,95	100	13,73	3
88	-7,45	193	18,2	26,39	31,83	82,96	12,07	3	87	-8,27	223,57	13,6	17,67	25,13	69,64	9,51	3

96	-10,21	300,86	3,5	21,08	21,74	96,96	14,13	2,75	87,7	-6,43	273	1	18,26	20,4	89,4	11,71	2,58
69	-8,05	228	14,3	19,44	19,44	100	11,42	3	86	-6,16	78,21	17,6	21,32	21,32	100	13,76	3
77,9	-9,57	249,43	27,3	15,72	16,05	97,22	10,86	2,83	77	-7,43	177	32,3	16,02	16,31	97,96	12,09	3
72	-6,25	214,29	11,8	19,63	19,63	100	11,92	3	76,3	-12,66	297,03	7,2	19,63	19,63	100	11,92	3
66	-4,85	95,45	42,3	18,88	23,45	80,78	11,54	3	76	-9	184,64	27,6	18,88	23,45	80,78	11,54	3
87	-7,53	155,64	20,8	18,63	24,39	76,57	8,38	3	94	-9,63	315	11,7	18,63	24,39	76,57	8,38	3

StO2_D 4	Downslo p	Upslope _	DAUC_D 4	LOS_IC U	ICU_Mo rt	aAPACHE_ a	NE_mcg/ k	HR_D1	MAP_D 1	HB_D1	Transfusi o	Age	MAP_D 2	HR_D2	NE_D2	Transfusi o	Hb_D2
77,8	-5,35	105	25,8	15	0	26	0,96	83	90	10,2	1	78	71	112	0,46	0	9,7
73	-1,84	63,27	37,3	24	0	13	0,21	74	95	10,4	0	75	90	86	0,28	0	8,6
75	-10,68	113,18	24,7	17	0	17	0,24	95	96	10,3	0	56	85	60	0,24	1	8,3
86	-7,45	106,09	5,8	54	0	9	0	77	83	11,3	0	88	69	75	0	0	12,1
				28	0	20	0,05	56	66	10,9	0	33		63	0,08	0	9,9
83	-10,33	132,5	0	12	0	11	0,62	94	68	10,2	0	40	95	129	0,62	1	8,1
77	-9,07	112,91	7,2	7	0	16	0,03	45	85	13,9	0	26	115	45	0,02	0	13
78	-10,25	295,71	28,3	26	0	14	0,08	62	84	10,4	0	18	94	66		0	9,8
83	-6,26	190,5	19,4	10	0	14	0	117	123	9,8	0	47	89	84	0,12	1	9,9
78	-6,64	222	10,6	17	0	12	0,48	74	87	11,3	0	23	82	59	0,29	1	8,9
90	-6,74	247,72	7,2	15	0	15	0,3	102	75	8,1	1	44	56	101	0	1	8,6
83	-9,11	126,92	7,2	9	0	5	0,3	105	77	10,9	1	40	98	96	0,22	0	8,2
75	-9,41	211,07	24,8	5	0	17	0,17	116	98	13,3	0	86	100	130	0,17	0	12,6
75,9	-12,12	204,86	12,3	7	0	21	0,18	52	92	11,1	1	81	109	133	0,18	0	10,9
71,5	-16,1	84,21	14,2	45	0	23	0,8	50	94	8,8	1	73	81	83	0,47	0	10
72	-14,58	177	0	7	0	18	0	66	100	12,3	0	44	99	60	0	0	11,2
72	-11,44	77,5	0,1	26	0	17	0	61	62	11,3	0		68	76	0,08	0	10,7
86	-8,35	213,21	23,8	13	0	21	0	61	104	9,6	1	78	100	63	0	0	9,4
68,2	-9,38	93,82	18,8	5	0	14	0,58	100	58	10,5	1	71	78	65	0,67	0	9
82	-12,12	283	8,2	4	0	4	0	116	102	8,8	1	31	67	110	0	0	9,1
74,8	-9,68	194,57	11,8	11	0	11	0	62	78	9,6	1	66	68	70	0	0	9,4
85	-7,92	294,86	19,5	15	0	9	0	78	80	12,7	0	68	100	78	0	0	12,5
73	-4,54	255	17,2	5	0	16	0,13	64	88	8,8	0	77	92	105	0,13	0	9,1
92	-5,18	144	0,1	6	0	16	0,22	56	84	12,6	0	71	82	58	0,13	0	12,5
80	-6,56	169	28	7	0	1	0	85	74	12,5	0	18	81	82	0	0	11,6
76,3	-12,66	297,03	7,2	4	0	0	0	106	89	11,1	0	32	85	85		0	9,5
76	-9	184,64	27,6	4	0	7	0	74	90	11,3	1	54	95	94	0	0	10,9
94	-9,63	315	11,7	4	0	14	0	52	70	12,1	0	72	78	49	0	0	10,8

MAP_D3	HR_d3	NE_D3	Transfusio	Hb_D3	MAP_D4	HR_D4	NE_D4	Transfusio	Hb_D4
88	93	0,33	0	11,5	88	73	0,17	0	10,9
114	114	0,4	0	10,5	88	109	0,36	1	10
89	45	0,08	0	9,5	102	75	0,09	0	9,9
77	85	0	0	9,8	89	90	0,11	0	10,9
76	54	0,04	0	9	117	65	0,17	0	9,3
88	92	0,62	0	9,7	84	85	0,69	0	8,7
79	63	0,04	0	12,6	87	81	0,07	0	12
86	70	0,13	1	10,9	86	70	0,13	0	9,4

98	74	0,12	0	9,8	80	64	0,04	0	9,4
79	78	0,11	1	9,8	96	74	0,1	0	9,7
78	91	0	1	7,9	77	70	0	1	10,5
97	53	0,09	0	10,9	88	52	0,04	0	8,3
77	93	0,09	0	10,9					
103	70	0	0	10,5	97	68	0	0	10,2
90	91	0,44	0	9,4	55	87	0,31	1	8,4
104	58	0	0	11,9	104	60	0	0	12,4
64	68	0,04	0	9,1	75	72		0	8,7
95	71	0	0	9	109	63	0	0	9
67	60	0	1	8,9	70	63	0	1	8,2
83	117	0	0	9,2	84	84	0	1	7,9
88	60	0,05	0	8,8	81	73	0	0	8,2
97	76	0	0	12,6	120	85	0	0	13,1
103	120	0	1	6,7	107	120	0	0	9,9
87	71	0	0	11,5	104	101	0	0	11,6
82	78	0	0	11,2	73	77	0	0	10,6
90	86	0	0	9,2					
74	94	0	0	8,4					
108	65	0	0	10,6					

SOFA= Sequential Organ failure Score; PVDs= perfused small vessel density; TVDs= total small vessel density; PPV= percentage of perfused vessels; MFI= microvascular flow index; StO2= tissue oxygen saturation; AUC StO2= area under the curve of tissue oxygen saturation; LOS= Length of Stay; ICU= Intensive Care Unit; APACHE= Acute Physiology, Age, Chronic Health Evaluation; NE=Norepinephrine; HR= Heart Rate; MAP= Mean Arterial Pressure; Hb= Haemoglobin.
